# Density of aortopulmonary collaterals predicts in-hospital outcome in tetralogy of Fallot with pulmonary stenosis

**DOI:** 10.1093/icvts/ivab238

**Published:** 2021-09-20

**Authors:** Yibing Fang, Ziqing Xiong, Yue Wang, Bo Li, Zetao Wang, Deying Kang, Qi An, Ke Lin, Shuhua Luo

**Affiliations:** Department of Cardiovascular Surgery, West China Hospital of Sichuan University, Chengdu, China; Department of Cardiovascular Surgery, West China Hospital of Sichuan University, Chengdu, China; Department of Cardiovascular Surgery, West China Hospital of Sichuan University, Chengdu, China; Department of Radiology, West China Hospital of Sichuan University, Chengdu, China; Department of Radiology, West China Hospital of Sichuan University, Chengdu, China; Department of Evidence‐based Medicine and Clinical Epidemiology, West China Hospital of Sichuan University, Chengdu, China; Department of Cardiovascular Surgery, West China Hospital of Sichuan University, Chengdu, China; Department of Cardiovascular Surgery, West China Hospital of Sichuan University, Chengdu, China; Department of Cardiovascular Surgery, West China Hospital of Sichuan University, Chengdu, China

**Keywords:** Tetralogy of Fallot, Aortopulmonary collateral artery, Outcome

## Abstract

**OBJECTIVES:**

The aim of this study was to characterize the anatomy of aortopulmonary collateral (APC) arteries in tetralogy of Fallot and pulmonary stenosis and to determine whether APC density identified on preoperative multidetector cardiac computed tomography predicts in-hospital outcome.

**METHODS:**

The retrospective single-centre study includes consecutive 135 (2015–2019) patients who underwent one-stage repair. Preoperative multidetector cardiac computed tomography, echocardiography and clinical outcomes were reviewed. The cut-off value of indexed total distal APC cross-sectional area (APC-CSA) was identified by receiver operating characteristic curve. Logistic regression was used for predictors analysis.

**RESULTS:**

The median age and body weight were 19.7 (10.1–89.7) months and 10 (8.3–18) kg. A total of 337 APCs were detected with only one demonstrating severe stenosis. There was a strong and significant correlation between mean APC diameter per patient and age (*r* = 0.70, *P* < 0.001). APCs were imaged but mainly received no interventions. In-hospital mortality was similar between patients with high (indexed APC-CSA **≥**3.0 mm^2^/m^2^) and low (**<**3.0 mm^2^/m^2^) APC density (*P* = 0.642). Significantly greater patients with high indexed APC-CSA experienced the in-hospital composite outcome of death, arrest, renal/hepatic injury, lactic acidosis or extracorporeal membrane oxygenation (*P* = 0.007). High APC density was associated with greater dosing (*P* = 0.008) and longer (*P* = 0.01) use of inotropic support, prolonged pleural drainage (*P* = 0.013), longer ventilation (*P* = 0.042), intensive care unit (*P* = 0.014) and hospital (*P* = 0.027) duration. No reintervention and death occurred in the follow-up with the median duration of 24.4 (11–36.6) months. Multivariable analysis showed the Nakata index (*P* = 0.05) and high APC density (*P* = 0.02) independently predicted the composite outcome.

**CONCLUSIONS:**

In tetralogy of Fallot and pulmonary stenosis, APCs are likely to be dilated bronchial arteries. Preoperative multidetector cardiac computed tomography-derived APC density was as important as Nakata index in predicting the occurrence of in-hospital composite outcome. The APC-CSA of 3.0 mm^2^/m^2^ maybe considered as a threshold for risk stratification.

**Figure:**
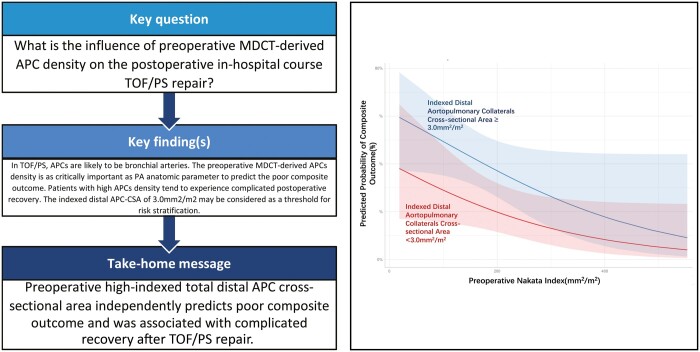

## INTRODUCTION

The detailed anatomic characteristics of aortopulmonary collateral (APC) arteries in patients with the tetralogy of Fallot and pulmonary stenosis (TOF/PS) remain unclear. The evidence linking APCs to postoperative recovery is scarce. The presence of APCs has been suspected to result in unfavourable postoperative haemodynamics [1]. However, previous studies failed to show the impact of the amount of APCs on early postoperative outcome [1–3].

Multidetector cardiac computed tomography (MDCT) yields precise preoperative information regarding the detailed anatomy of the APCs [4] with a marked decrease in the radiation exposure. The objectives of the present study were to characterize the anatomy of APCs in TOF/PS and to investigate the influence of preoperative MDCT-derived APC density on the postoperative in-hospital course. To avoid confusion in nomenclature, we have used the term APCs to collectively refer to all arteries branching from the systemic circulation and entering the lungs, irrespective of their size, origin or number.

## METHODS

We retrospectively reviewed all patients who underwent TOF/PS repair between October 2015 and December 2019. The Research Ethics Board at West China Hospital approved the study and waived the requirement for patient consent. Patients with prior cardiac surgery, TOF with pulmonary atresia, TOF with absent pulmonary valve and TOF with atrioventricular canal defects were excluded. A total of 135 consecutive patients were included in present study.

### Preoperative multidetector cardiac computed tomography scan

In addition to echocardiography, MDCT is routinely performed for preoperative assessment of the pulmonary arteries (PAs), APCs and coronary arteries. This is because near one-fifth of patients at our institution were adult whose acoustic windows of echocardiography are usually poor. Two (2/135, 1.5%) patients also underwent preoperative catheterization. Helical computed tomography angiography was performed on a single-detector scanner (256-slice, GE Revolution Healthcare). The scanning techniques were adapted to patient weight with the ‘as low as reasonably achievable’ principle. We usually use 1 dose of 1.5 ml/kg (maximal of 80 ml) non-ionic, low-osmolar contrast agent (Iomeprol 400; Patheon Italia S.P.A.) with a concentration of 300 mg I/ml for paediatric patients. The images were reconstructed onto the mediastinum using the zoom feature with a field of view of 220 mm and matrix 512 × 512. The region of interest involving the aorta and its main branches was outlined. The helical scanning with breath-hold automatically start when region of interest reaching 120 Hu. To reduce the risk of breathing artefacts, we chose to scan in a caudocranial direction.

### Analysis of multidetector cardiac computed tomography images

The obtained axial images were transferred to a workstation (AW4.6, GE Medical Systems, Milwaukee, WI, USA) for processing and analysis. MDCT images were evaluated by 2 senior cardiovascular radiologists (B.L. and Z.W.), and decisions were made in consensus. The anatomy of APCs was assessed using the methods established in previous studies of bronchial arteries (BAs) anatomy in normal population [5] and patients with TOF/pulmonary atresia/ventricular septal defect (VSD) [6]. The origin of each APC was reported as intercostal trunk (ICT), common trunk or directly from the aorta. The proximal diameter of APC was measured at immediately after its point of origin. Each APC was followed down to the hila for measurement of the distal diameter. The anatomy of APCs was analysed by recording the following parameters: (i) the side (left versus right); (ii) the proximal and distal diameter; (iii) the origin; (iv) the site of the APC ostium (orthoptic versus ectopic); and (v) the mediastinal course in relation to the oesophagus (on dorsal or ventral side). Other parameters such as the diameter of the branch PA and main PA, and the laterality of aortic arch were also recorded.

### Surgical techniques

The median time between preoperative MDCT and the operation was 7 (3–11) days. Primary repair was considered in patients at 3–6 months of age. We usually proceed with palliation for symptomatic patients (saturation < 80%) younger than 3 months. Only 2 patients had APC embolization preoperatively. Reparative techniques were similar for all patients, and no concomitant surgical APC ligation was performed in the current cohort. Cardiopulmonary bypass (CPB) was initiated via bicaval cannulation with mild hypothermia (34°C). Myocardial protection was achieved by antegrade cold blood cardioplegia repeated at 25-min interval. Intraoperative transoesophageal echocardiography was routinely performed to document the adequacy of the repair. Transannular patch was considered if (i) *Z*-score of PA annulus <−2 or (ii) right ventricular systolic pressure/left ventricular systolic pressure >0.7 post-repair.

### Data collection

Patients’ demographic data, perioperative variables, MDCT-derived anatomic characteristics of APCs, catheterization, echocardiographic data and the intraoperative information were collected. Given that death occurred rarely in our study population, our choice of a primary outcome was a composite morbidity–mortality outcome consisting of 6 individual criteria selected based on similar studies looking at the outcomes after paediatric cardiac surgery [7, 8]. Patients experienced any of the 6 components post-repair and before hospital discharge were considered to have met the composite outcome. The components are death, cardiac arrest, postoperative extracorporeal membrane oxygenation, hepatic injury and renal insufficiency (creatinine >2 times the upper limited of normal) and lactic acidosis (>5 mmol/l). Hepatic injury was defined as previously reported [8]. Secondary outcomes include the maximal vasoactive inotropic score calculated in the first 24 h after intensive care unit (ICU) admission, the duration of inotropic support, the requirement of delayed sternal closure, duration of mechanical ventilation, length of ICU stay and hospitalization and prolonged chest tube insertion (the duration of chest tube ≥4 days).

### Statistical analysis

Continuous variables were expressed as a median and interquartile range (1st and 3rd quartiles) [9] and categorical variables were expressed as absolute numbers with percentages. The mean diameter of APCs was calculated using the proximal diameter of APCs (*D*) and the number of APCs in each patient according to the following formula: mean APC diameter = ∑*D*/*n*. The correlation between mean APC diameter and other variables was measured using the Spearman’s rank correlation. Two patients who received preoperative transcatheter APC embolization were excluded in the analysis of clinical outcome. The cross-sectional area (CSA) of the different APCs in each patient were summed to yield the total CSA of the APCs at proximal and distal point respectively. The cut-off point of the indexed total distal APC-CSA for the occurrence of composite outcome was identified by receiver operating characteristic curve, and patients were then divided into 2 groups of high (**≥**3.0 mm^2^/m^2^) and low (**<**3.0 mm^2^/m^2^) APC density determined by calculated distal APC-CSA cut-off point. The *t*-test, Mann–Whitney test and Fisher’s exact test were used to compare the 2 groups as appropriate. A univariable and multivariable logistic regression model was used to assess the association between preoperative covariates and composite outcome. The covariates with *P*-value of <0.8 in the univariable analysis were selected for the final multivariable model. Statistical analysis was performed with Stata Statistical Software (Release 14; StataCorp LP, College Station, TX, USA) and R version 3.6.3 (R Foundation for Statistical Computing, Vienna, Austria).

## RESULTS

### Anatomic characteristics of aortopulmonary collaterals

A total of 337 APCs were detected in 135 patients (Supplementary Material, Table S1). The number of right and left APCs was similar. The majority of APC ostium sites was orthotopic (78.2%) (Supplementary Material, Fig. S1A). The most common sites of ectopic APC ostium were at the aortic arch (54.9%), followed by subclavian artery (32.9%) (Supplementary Material, Fig. S1B and C). Around one-third of APCs (35.3%) originated from aorta directly, the rest of them arose from the aorta as a common trunk (41.6%) and an ICT (23.1%) (Supplementary Material, Fig. S1D–F). All of the 337 APCs coursed along the ventral side of oesophagus. The proximal diameter of the majority of APCs (75.9%) was smaller than 2 mm. The distal diameter of APCs was significantly smaller than the proximal diameter (*P* = 0.00). Only one APC showed proximal stenosis ≥70%.

### Preoperative parameters and assessment of aortopulmonary collaterals

Patients’ demographic information and APC characteristics are summarized in Supplementary Material, Table S2. Patients’ median age and weight were 19.7 (10.2–89.7) months and 10 (8.3–18.0) kg, respectively. More than half (62.1%) of patients received repair at the age younger than 3 years. The median APC number per patient was 3 (2, 3) with the majority (80.0%) <4. Indexed distal APC-CSA was significantly smaller than indexed proximal APC-CSA (*P* = 0.00), and there was a significant positive correlation between the 2 (*r* = 0.47, *P* = 0.00) (Supplementary Material, Fig. S2).

Supplementary Material, Table S3 shows the variation in the APC branching pattern. A total of 12 branching patterns were identified in the current cohort, and 5 (3.7%) patients did not have orthotropic APCs. The most 2 common patterns were ICT (34.1%) and common trunk (28.1%) with or without direct APC, followed by the combination of 1 ICT and 1 common trunk (20.7%). ICT-type branching patterns were more common in patients with left arch (*P* = 0.004), whereas common trunk-type branching patterns were more common in patients with right arch (*P* = 0.001).

### The correlation between characteristics of aortopulmonary collaterals and patients’ parameters

**Figure 1:**
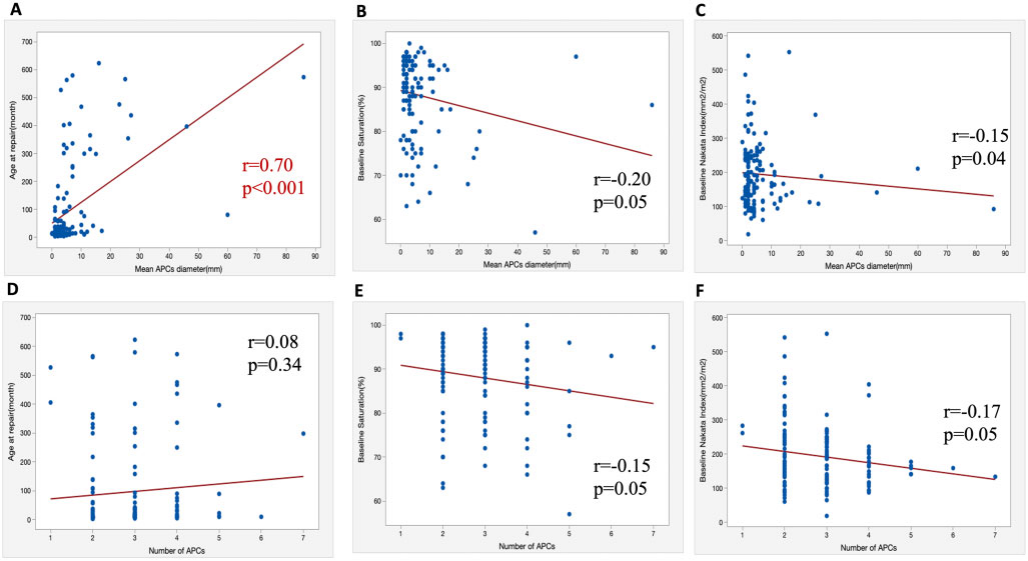
The correlation between characteristics of aortopulmonary collaterals and patient parameters. The mean diameter of APCs was calculated using the prxoimal diameter of APCs (*D*) and the number of APCs in each patient according to the following formula: mean APC diameter = ∑*D*/*n*. The mean APC diameter in each patient was strongly correlated with age (*r* = 0.70, *P* = 0.00) (**A**) and significantly correlated with preoperative Nakata index (*r* = −0.15, *P* = 0.04) (**B**) and arterial saturation (*r* = −0.20, *P* = 0.05) (**C**). The APC number (*n*) was negatively correlated with Nakata index (*r* = −0.17, *P* = 0.05) (**E**) and preoperative saturation (*r* = −0.15, *P* = 0.05) (**F**), respectively. APCs: aortopulmonary collaterals.

As shown in Fig. 1, the mean APC diameter was strongly correlated with age (*r* = 0.70, *P* < 0.001) and significantly correlated with preoperative arterial saturation (*r* = −0.20, *P* = 0.05) and Nakata index (*r* = −0.15, *P* = 0.04). The APC number was negatively correlated with preoperative saturation (*r* = −0.15, *P* = 0.05) and Nakata index (*r* = −0.17, *P* = 0.05), respectively. Of note, the strength relationships between APC size/number and preoperative saturation/Nakata index were very weak.

### Pre- and intraoperative characteristics between patients with high and low aortopulmonary collateral density

A total of 133 patients (2 patients were excluded due to the preoperative transcatheter APC embolization) were divided into high and low APC density groups based on the indexed distal APC-CSA cut-off value (3.0 mm^2^/m^2^) identified using the receiver operating characteristic curve (Supplementary Material, Fig. S3). As shown in Supplementary Material, Table S4, patients with a high APC density were significantly younger (*P* = 0.02), smaller body weight (*P* = 0.005) and smaller main and branch PA (*Z*-score of main PA, *P* = 0.04; Nakata index, *P* = 0.005; Mcgoon ratio, *P* = 0.0008) when compared to patients with a low APC density. As expected, both mean diameter of APCs (*P* = 0.004) and indexed proximal APC-CSA (*P* = 0.00) were significantly greater in the high APC density group. The intraoperative parameters were comparable between groups.

### Postoperative outcome

A total of 41 patients (30.8%) met the criteria of composite outcome (Table 1), and the most 2 common criteria met were hepatic injury (27.1%) and lactic acidosis (20.3%). There were 3 in-hospital deaths. Two deaths were cardiac related: 1 patient had persistent malignant ventricular arrhythmia immediately after extubation on postoperative day 2 and another patient expired due to refractory hypotension, bradycardia and severe heart failure at postoperative day 0. Another patient died from multiple organ failure at postoperative day 5 due to massive postoperative gastrointestinal bleeding. The incidence of mortality was similar between the 2 groups (*P* = 0.64); however, the occurrence of composite outcome was significantly more common in the high APC density group (*P* = 0.007). In addition, patients with a high APC density experienced complicated postoperative recovery requiring greater inotropic support (maximal vasoactive inotropic score, *P* = 0.008; duration of inotropic support in hours, *P* = 0.04), longer chest tube insertion (*P* = 0.01), longer ICU duration (*P* = 0.01) and overall hospital stay (*P* = 0.03).

**Table 1:** Comparison of in-hospital outcome between low and high aortopulmonary collaterals density

Variable	Sum (*n* = 133)	Low APC density (APC-CSA <3.0 mm^2^/m^2^, *n* = 62)	High APC density (APC-CSA ≥3.0 mm^2^/m^2^, *n* = 71)	*P*-value
Composite outcome (*n*)	41 (30.8%)	12 (19.4%)	29 (40.8%)	0.007
Death (*n*)	3 (2.3%)	1 (1.6%)	2 (2.8%)	0.642
Circulatory support (*n*)	1 (0.7%)	1 (1.6%)	0 (0%)	0.225
Cardiac arrest (*n*)	5 (3.8%)	1 (1.6%)	4 (5.6%)	0.284
Renal insufficiency (*n*)	5 (3.8%)	2 (3.2%)	3 (4.2%)	0.741
Hepatic injury (*n*)	36 (27.1%)	11 (17.7%)	25 (35.2%)	0.024
Lactic acidosis (*n*)	27 (20.3%)	12 (19.4%)	15 (16.9%)	0.080
Delayed chest closure (*n*)	6 (4.5%)	1 (1.6%)	5 (7.0%)	0.121
Maximal VIS in the first 24 h after ICU admission	9 (5–20)	7 (3–18)	10 (6–20)	0.008
Duration of vasoactive support (h)	77 (40–142)	48 (23–139)	96 (61–161.5)	0.010
Ventilation time (h)	20 (5–91)	11 (4–67)	30 (6–119)	0.042
ICU duration (days)	5 (3–8)	4 (2–7)	6 (3–9)	0.014
Hospital stay (days)	9 (7–13)	8 (7–12)	10 (8–15)	0.027
Prolonged chest insertion (*n*)	35 (26.3%)	10 (16.1%)	25 (35.2%)	0.013
Postoperative catheterization (*n*)	7 (6.0%)	2 (3.2%)	6 (8.5%)	0.208

APCs: aortopulmonary collaterals; CSA: cross-sectional area; ICU: intensive care unit; VIS: vasoactive inotropic score.

The clinical characteristics of patients (*n* = 8) who underwent postoperative catheterization are summarized in Supplementary Material, Table S5. The majority of catheterization procedures (7/8, 87.5%) was the embolization of enlarged APCs mainly due to postoperative respiratory distress (*n* = 6). Among patients receiving APC embolization, 6 out of 7 (85.7%) were preoperatively categorized into the high APC density group, and the left atrium pressure measured in these patients was higher than 20 mmHg.

No reintervention and death occurred in the follow-up period, with the median duration of 24.4 (11–36.6) months.

### Predictors for the composite outcome

**Figure 2:**
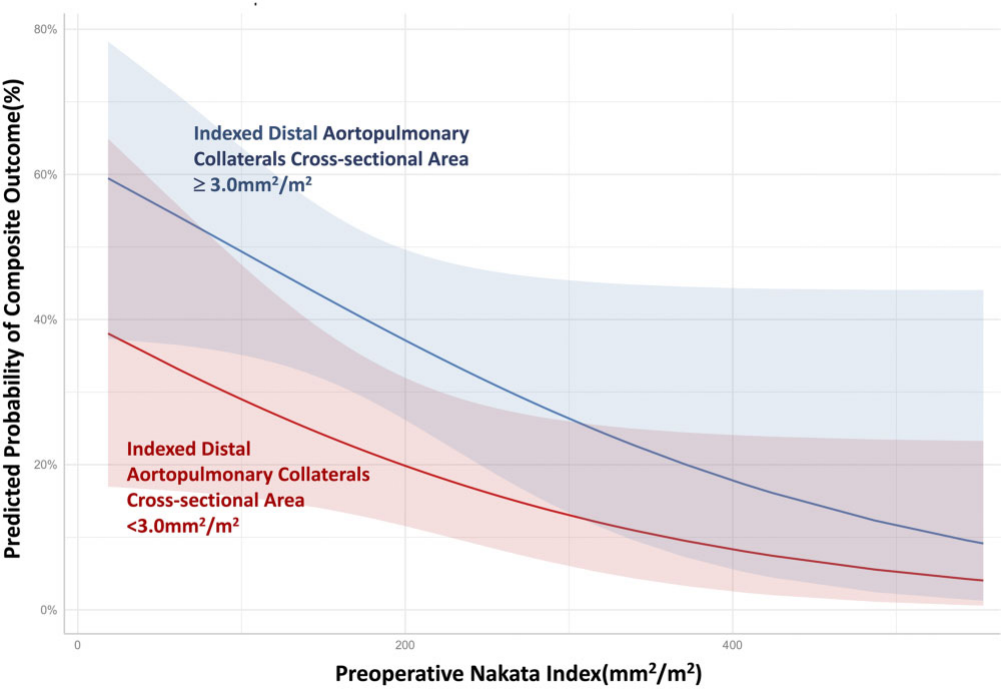
Predictors for the in-hospital composite outcome. The multivariable analysis showed APC density [high versus low aortopulmonary collaterals density: coefficient, 0.950 (0.141, 1.758), *P* = 0.02] and Nakata index [coefficient, −0.72 (−0.204, 0.07), *P* = 0.05] as independent predictors for the composite outcome consisting of 6 individual criteria including death, cardiac arrest, postoperative extracorporeal membrane oxygenation, hepatic injury and renal insufficiency and lactic acidosis.

**Figure 3:**
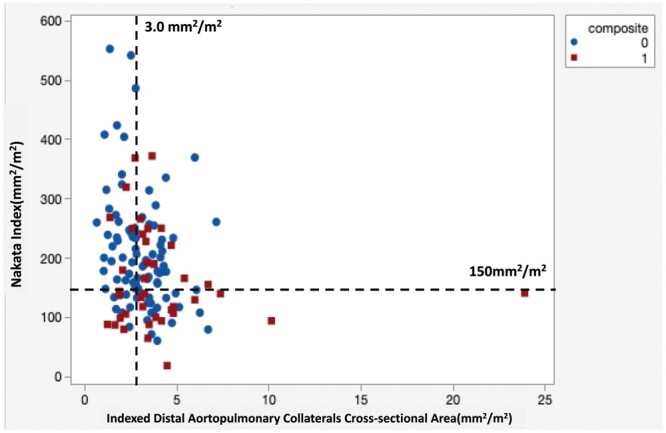
The relationship between occurrence of composite outcome, preoperative aortopulmonary collaterals density and Nakata index. The occurrence of the composite outcome consisting of 6 individual criteria including death, cardiac arrest, postoperative extracorporeal membrane oxygenation, hepatic injury and renal insufficiency and lactic acidosis was more common in patients with indexed aortopulmonary collateral-cross-sectional area >3.0 mm^2^/m^2^, and Nakata index smaller than 150 mm^2^/m^2^.

As shown in Table 2, the univariable logistic regression model showed that low APC density was significantly associated with lower risk of composite outcome [odds ratio 2.876 (1.308–6.326), *P* = 0.009]. Conversely, lower Nakata index [odds ratio 0.993 (0.988–0.998), *P* = 0.012] and smaller main PA [odds ratio 0.884 (0.777–1.005), *P* = 0.062] were associated with higher risk of composite outcome. Age (*P* = 0.710) and proximal APC-CSA (*P* = 0.09) were not associated with the composite outcome. The multivariable analysis showed high APC density [odds ratio 2.585 (1.152–5.800), *P* = 0.02] and low Nakata index [odds ratio: 0.460 (0.206–1.028), *P* = 0.05] as independent predictors for the composite outcome (Fig. 2). The occurrence of the composite outcome was more common in patients with indexed APC-CSA >3.0 mm^2^/m^2^ and Nakata index smaller than 150 mm^2^/m^2^ (Fig. 3).

**Table 2:** Univariable and multivariable logistic regression on predictors associated with composite outcome

Variable	Univariable odds ratio	Univariable *P*-value	Multivariable odds ratio	Multivariable *P*-value
High versus low indexed distal APC-CSA (ref: low)	2.876 (1.308, 6.326)	0.009	2.585 (1.152, 5.800)	0.02
Proximal APC-CSA (mm^2^/m^2^) (for each 0.1 mm^2^/m^2^ increase)	1.054 (0.996, 1.115)	0.09		
Distal APC-CSA (mm^2^/m^2^) (for each 0.1 mm^2^/m^2^ increase)	1.300 (1.016, 1.665)	0.03		
Weight (kg) (for each 1 mm^2^/m^2^ increase)	0.978 (0.952, 1.005)	0.114		
Age (months) (for each 1 month increase)	0.999 (0.996, 1.002)	0.710		
Nakata index (mm^2^/m^2^) (for each 0.1 mm^2^/m^2^ increase)	−0.973 (−1.730, −0.215) 0.993 (0.988, 0.998)	0.012	0.460 (0.206, 1.028)	0.05
Z-score of main PA (for each 1 increase)	0.884 (0.777, 1.005)	0.062	0.930 (0.811, 1.067)	0.30
Indexed LVEDV (ml/m^2^) (for each 0.1 ml/m^2^ increase)	1.010 (0.982, 1.038)	0.465		
Indexed RVEDV (ml/m^2^) (for each 0.1 ml/m^2^ increase)	1.040 (0.992, 1.090)	0.102		

APCs: aortopulmonary collaterals; CSA: cross-sectional area; LVEDV: left ventricular end-diastolic volume; PA: pulmonary artery; RVEDV: right ventricular end-diastolic volume.

## DISCUSSION

The current study was undertaken to characterize the anatomy of APCs in TOF/PS patients and to determine whether preoperative MDCT-derived APC density predicts postoperative in-hospital composite outcome. We fully recognized that the median age of our patients undergoing repair was greater than the typical practice in most developed countries. This is a reflection of the fact that patients in developing countries usually do not receive timely repair due to limited paediatric medicine infrastructure and a lack of specialized medical centres [10]. However, our patients population provided a unique opportunity to analyse the APC anatomy over broad age ranges. The anatomic characteristics of APCs were substantially different to major aortopulmonary collateral arteries (MAPCAs), indicating that these collaterals are likely to serve as BAs in patients with TOF/PS. High APC density independently predicts the occurrence of in-hospital composite outcome. Furthermore, patients with a high APC density tend to have a complicated postoperative recovery. These findings suggested that the preoperative APC density is as critically important as Nakata index in predicting the in-hospital composite outcome. The indexed distal APC-CSA of 3.0 mm^2^/m^2^ may be considered as a threshold for risk stratification. A more proactive preoperative APC embolization may be considered in TOF/PS patients with high APC density based on current findings. The associated benefit and risk require further evaluation in the future prospective study. Nevertheless, we believe that the use of MDCT should be reserved for the patients with significant small branch PA and/or multiple APCs shown on preoperative echocardiography.

### Aortopulmonary collaterals may be bronchial artery in tetralogy of Fallot and pulmonary stenosis

The large APCs in TOF/pulmonary atresia/VSD patients, known as MAPCAs, are well described. The MAPCAs may persist beyond birth when the central PA system fails to develop proper continuity with the embryonic lung in TOF/pulmonary atresia/VSD. MAPCAs frequently follow a retro-oesophageal course. The diameter of MAPCAs tends not to increase over time. Up to 60% of MAPCAs become significant stenotic with eventual occlusion and loss of lung segments if no other supply is available. Non-stenotic MAPCAs, on the other hand, perfuse the pulmonary vasculature under systemic pressure, which ultimately lead to obstructive pulmonary vascular disease. In patients with TOF/PS, APCs were often described by 2 interchangeable nomenclatures including enlarged BAs and MAPCAs in the literatures, highlighting the inadequate understanding of these vessels in uncomplicated TOF. APCs in the current study were shown to have a substantially different anatomic characteristics pattern from MAPCAs: APCs rarely develop significant stenosis and are not associated with lung segments loss or obstructive pulmonary vascular disease in our cohort. Furthermore, other anatomic characteristics of APCs including distribution of ostium site, nature of normal tapering, and relationship of mediastinal course to the oesophagus are similar to BAs. These findings suggested the APCs in TOF/PS are likely to be BAs as opposed to MAPCAs. Future biological comparative analysis such as gene expression may better explain the difference of morphology and growth pattern between MAPCAs and BAs.

### The variation of branching pattern of aortopulmonary collaterals

Previous studies revealed that the predominant BAs’ branching pattern is BAs arising from the descending aorta via an ICT in up to 75–90% of subjects [4, 5]. In contrast to previous studies, the occurrence of this anatomical feature in our cohort was relatively low (54.7%). This is particularly true in patients with right arch: only 9 patients (32.1%) had ICT. To our knowledge, the correlation between BA branching pattern and aortic arch variations has not been reported. Given that the most prominent segmental medullary artery, the artery of Adamkiewicz, mainly arises from an intercostal artery, this variation of APCs’ branching pattern with right arch may be important for the planning of APC embolization.

### Predictors associated with the composite outcomes

Previous studies showed that the presence of enlarged APCs was associated with prolonged hospital stay [1] or ventilation [3]. However, the current literature has yet to correlate the quantitative amount of APCs with early postoperative outcomes. Given that APCs appear to be very common in TOF, establishing cut-off point of APC density predicting unfavourable in-hospital outcome is warranted to guide the clinical practice. Some authors suggested that, based on their clinical observation, APC reduction should be performed in patients with an APC diameter-to-body weight ratio of >0.5 mm/kg [3] or diameter >2 mm [11]. However, the association between these criteria and postoperative outcome has not been validated. Consistent with previous study, our results confirmed the adverse effect of APCs on postoperative in-hospital recovery. Furthermore, we proposed a potential cut-off point for predicting the occurrence of in-hospital composite outcome based on the indexed distal APC-CSA.

The difference between groups in the composite outcomes seems to be driven mainly by the liver injury. This could be explained by the relatively small sample size, and low incidence of death (*n* = 3, 2.3%), circulatory support (*n* = 1, 0.7%) and renal insufficiency (*n* = 5, 3.8%) in the current cohort. Hepatic injury represents an important early clinical outcome and was considered as an indicator of poor prognosis in paediatric patients. Up to 20–30% of children experienced various levels of postoperative liver injury following cardiac surgery [8, 12]. Based on the pathophysiology, there are 2 different kinds of hepatic injury including ischaemic hepatitis (‘shock liver’), which is often attributed to profound hypotension or haemodynamic instability and passive liver congestion, which is usually associated with venous congestion secondary to the right heart failure [13]. The high APC density-related hepatic injury in the current study was likely to be the result of inadequate tissue oxygen delivery as the central venous pressure and CPB time were similar between the 2 groups.

Intriguingly, the proximal APC-CSA, although significantly correlated with the distal APC-CSA, did not predict the poor clinical outcome. In normal population, around 30% of bronchial circulation flow perfuses the extrapulmonary structures and drains into the right atrium. The rest of BAs flow supplies the intrapulmonary bronchi (and bronchioles) and joins the pulmonary veins to drain into the left atrium, forming the greater part of systemic-to-pulmonary shunt. This made the usage of proximal diameter of APCs as a surrogate for the amount of systemic-to-pulmonary flow controversial [14]. Some factors such as APCs’ length, which could potentially influence the amount of shunt flow, were not included in the analysis. Further study is needed to determine whether the distal APC-CSA is highly correlated with the amount of shunt measured by cardiac magnetic resonance imaging in TOF/PS patients, as in the case of chronic pulmonary thromboembolism [15].

As expected, and also consistent with previous studies, small Nataka index was associated with adverse in-hospital outcomes. Univariable analysis did not demonstrate low operative age and low weight as risk factors in this cohort, presumably due to the fact that there were few of such patients (age <6 months: *n* = 6; weight <5 kg: *n* = 1) in our cohort.

### Mechanisms related to the impact of aortopulmonary collaterals on the in-hospital outcome

APCs were identified as the source of a considerable systemic-to-lung shunt and consume a sizeable portion of the cardiac output [16]. We suspect the cumulative APC flow, at least in the early postoperative period, may decrease the cardiac output necessitating inotropic support and impair organ perfusion, causing lactic acidosis and hepatic. The left ventricle also appears to be fairly intolerant of the increased volume load from the shunt. This is suggested by the significantly elevated left atrium pressure in patients who received postoperative catheterization. Of note, the preoperative left ventricular size of these patients was not particularly small. Interestingly, patients with high APC density had longer duration of chest tube insertion. Also, among the patients who received postoperative catheterization, 4 out of 8 developed chest effusion. This result suggests that restrictive right ventricular physiology may play a potential role in the association of APCs with unfavourable in-hospital outcomes. We speculated that the high end-diastolic pressure of left ventricle may impair the right ventricular diastolic function via ventricular–ventricular interactions. On the other hand, restrictive right ventricular physiology may influence left ventricular filling, resulting in pronounced pulmonary venous reversals and larger left atrial size [17]. Further studies showing the relationship between APC flow and cardiac output and demonstrating diastolic function of both ventricles may provide further insight into the mechanisms behind the impact of APCs on postoperative outcomes.

### Limitations 

The major limitations of this study include the retrospective, observational nature of a study from a single institution. Discriminatory power of APC density for in-hospital composite outcome was low as quantified by the area under the receiver operating characteristic curve. This could be caused by the limited cohort size, and small number of patients with the unfavourable in-hospital outcome. Given the limited sample size, the paper should be positioned as a hypothesis-generating study. The independent association between high APC density and poor outcomes warranted to be verified in a large sample size study as the baseline patient’s characteristics were significantly different between high versus low APC density groups. Lastly, the follow-up period was not long enough to effectively conclude the fate of APCs after repair.

## CONCLUSIONS

In patients with TOF/PS, APCs are likely to be BA. The preoperative quantification of APCs by MDCT is as critically important as PA anatomic parameter in predicting the in-hospital composite outcome. Patients with significant APC burden tend to experience complicated postoperative recovery. The indexed distal APC-CSA of 3.0 mm^2^/m^2^ may be considered as a threshold for risk stratification.

## SUPPLEMENTARY MATERIAL

Supplementary material is available at *ICVTS* online.

**Conflict of interest:** none declared.

## Supplementary Material

ivab238_Supplementary_Data

## Notes

Presented at the CHSS & ECHSA Joint Meeting 2020 Virtual Annual Meeting, 24–25 October 2020.

## ABBREVIATIONS

APC: Aortopulmonary collateral
BAs: Bronchial arteries
CPB: Cardiopulmonary bypass
CSA: Cross-sectional area
ICT: Intercostal trunk
ICU: Intensive care unit
MAPCAs: Major aortopulmonary collateral arteries
MDCT: Multidetector cardiac computed tomography
PA: Pulmonary artery
TOF/PS: Tetralogy of Fallot and pulmonary stenosis
VSD: Ventricular septal defect
