# Supplementary material for: Density of aortopulmonary collaterals predicts in-hospital outcome in tetralogy of Fallot with pulmonary stenosis
Source: Interact Cardiovasc Thorac Surg. 2021 Sep 20;34(2):307–14. doi: 10.1093/icvts/ivab238 (PMC8766212; doi:10.1093/icvts/ivab238)
Supplement: ivab238_Supplementary_Data [file ivab238_supplementary_data.docx]

**SUPPLEMENTAL TABLE**

**Supplemental Table 1: Characteristics of aortopulmonary collaterals**

| **Parameters** | **Value** |
| --- | --- |
| **Number** | **377** |
| Right (n) | 198(52.5%) |
| Left (n) | 179(47.5%) |
| **Ostium site** |  |
| Orthotopic (n) | 295(78.2%) |
| Ectopic (n) | 82(21.8%) |
| Aortic arch | 45 |
| Distal descending aorta | 5 |
| Internal thoracic artery | 2 |
| Subclavian artery | 27 |
| innominate artery | 3 |
| **Origin** |  |
| Separate (n) | 133(35.3%) |
| Common trunk (n) | 157(41.6%) |
| Intercostal trunk (n) | 87(23.1%) |
| **Dimension** |  |
| Distal diameter (mm) | 1.4(1.2, 1.8) |
| Proximal diameter (mm) | 1.5(1.3, 1.9) |
| < 2mm (n) | 286(75.9%) |
| 2-4mm (n) | 81(21.5%) |
| ≥ 4mm (n) | 10(2.7%) |
| **Proximal stenosis ≥70% (n)** | 1(0.27%)` |

**Supplemental Table 2: Preoperative assessment**

| **Characteristics** | **Value** |
| --- | --- |
| **Patient Number (n)** | 135 |
| **Male (n)** | 81(60.0%) |
| **Age (month)** | 19.7(10.1, 89.7) |
| 3 month-1 year (n) | 46 (34.0%) |
| 1-3 years (n) | 38(28.1%) |
| 3-18 years (n) | 27(20.0%) |
| >18 years (n) | 24(17.7%) |
| **Weight (kg)** | 10(8.3, 18) |
| **Presence of PDA (n)** | 14(10.4%) |
| **Presence of right arch (n)** | 28(20.7%) |
| **Preoperative saturation (%)** | 91(84, 95) |
| **Number of APCs (n)** | 3(2, 3) |
| ≤ 3 | 108(80.0%) |
| 4 - 5 | 25(18.5%) |
| ≥ 6 | 2(1.5%) |
| **APCs mean diameter (mm)** | 1.6(1.3, 1.9) |
| **Proximal APC-CSA (mm^2^)** | 4.3(2.8, 6.9) |
| **Distal APC-CSA (mm^2^)** | 1.5(1.1, 2.3) |
| **Indexed proximal APC-CSA (mm^2^/m^2^)** | 8.5(5.2, 12.8) |
| **Indexed distal APC-CSA (mm^2^/m^2^)** | 3.1(2.2, 4.0) |
| **McGoon ratio** | 1.52(1.81, 2.12) |
| **Nakata index** | 177.3(132.7, 242.1) |

APCs: Aortopulmonary collaterals; CSA: cross-sectional area;

**Supplemental Table 3：Variations in branching pattern of the aortopulmonary collaterals**

| **Branching patterns** | **Sum**  **(n=135)** | **Left arch(n=107)** | **Right arch(n=28)** | ***P*-value** |
| --- | --- | --- | --- | --- |
| **ICT(n)** | **46(34.1%)** | **43(40.2%)** | **3(10.7%)** | **0.004** |
| 1 ICT+1 contralateral APC | 32(23.7%) | 31(30.0%) | 1(3.6%) |  |
| 1 ICT+1 ipsilateral APC | 1(0.7%) | 1(0.9%) | 0 |  |
| 1 ICT | 8(5.9%) | 7(6.5%) | 1(3.6%) |  |
| 1 ICT+1 right APC+1 left APC | 5(3.7%) | 4(3.7%) | 1(3.6%) |  |
| **1 ICT+ 1 Common Trunck** | **28(20.7%)** | **22(20.6%)** | **6(21.4%)** | **0.552** |
| **Common Trunck(n)** | **38(28.1%)** | **23(21.5%)** | **15(53.6%)** | **0.001** |
| 1 common trunck | 31(23.0%) | 20(18.7%) | 11(39.3%) |  |
| 1 common trunck +1 APC | 6(4.4%) | 2(1.8%) | 4(14.2%) |  |
| 1 common trunck +1 left APC+1 right APC | 1(0.7%) | 1(0.9%) | 0 |  |
| **Separate(n)** | **18(13.3%)** | **14(13.1%)** | **4(14.3%)** | **0.539** |
| 1 right APC + 1 left APC | 13(9.6%) | 10(9.3%) | 3(10.7%) |  |
| 2 APC + 1 contrallateral APC | 2(1.4%) | 2(1.8%) | 0 |  |
| 1 APC | 3(2.2%) | 2(1.9%) | 1(3.6%) |  |
| **No orthotropic APCs** | **5(3.7%)** | **5(4.7%)** | **0** | **0.300** |

ICT: intercostal trunk; APCs: aortopulmonary artery

**Supplemental Table 4: Comparison of patient characteristics between low and high aortopulmonary collaterals density**

| **Variable** | **Sum(n=133)** | **Low APC density(APC-CSA <3.0 mm^2^/m^2^, n=62)** | **High APC density (APC-CSA ≥3.0mm^2^/m^2^, n=71)** | ***P*-value** |
| --- | --- | --- | --- | --- |
| **Preoperative parameters** | | | | |
| Age (month) | 19.7(10.1, 75.7) | 27.9(11.7, 107.5) | 14.5(9.0, 44.2) | 0.025 |
| Male (n) | 80(60.1%) | 42(67.7%) | 38(53.5%) | 0.096 |
| Weight (kg) | 10(8.3, 17.5) | 11 (9.0, 27) | 9.0(7.6, 15) | 0.005 |
| Right arch (n) | 27(20.3%) | 14 (22.6%) | 13(18.3%) | 0.542 |
| Presence of PDA(n) | 14(10.5%) | 7(11.3%) | 7(9.9%) | 0.789 |
| Preoperative saturation (%) | 91(84, 95) | 92.5(85, 95) | 90(78, 95) | 0.173 |
| Preoperative Hgb (g/L) | 141 (128, 169) | 141(127, 170) | 142 (128, 169) | 0.926 |
| Z -score of Main PA | -2.4(-4.2, -0.6) | -1.9(-3.7, 0) | -2.7(-4.6, -1.3) | 0.046 |
| Nakata index (mm^2^/m^2^) | 177.3(132.8, 242.1) | 203.7(143.9, 260.1) | 165.4(117.2, 221.7) | 0.005 |
| Mcgoon ratio | 1.82(1.52, 2.12) | 2.0(1.7, 2.3) | 1.7(1.4, 2.0) | 0.0008 |
| LVEDVI (ml/m^2^) | 44.7(37, 53.2) | 43.7(36.7, 53.8) | 46.3(37.2, 53.2) | 0.443 |
| RVEDVI (ml/m^2^) | 26(20, 32) | 23.5(19, 29) | 27 (22, 33) | 0.005 |
| Mean APC diameter(mm) | 1.6(1.3, 1.8) | 1.4(1.2, 1.8) | 1.6(1.4, 1.9) | 0.004 |
| Indexed proximal APC-CSA(mm^2^/m^2^) | 8.46(6.3, 10.8) | 6.3(4.4, 9.7) | 10.8(7.7, 13.7) | 0.000 |
| Indexed thickness of RV free wall (mm/m^2^) | 10.2(8.4, 12.8) | 10.0(8.1, 12.2) | 10.5(8.9, 13.1) | 0.208 |
| **Intraoperative parameters** | | | | |
| Cross clamp time (min) | 85(67.5, 99.5) | 80(63, 100) | 89(72, 98) | 0.277 |
| CPB time (min) | 120(102, 142.5) | 115(96, 140) | 126(104, 146) | 0.262 |
| Main PA pressure after repair (mmHg) | 23(18, 30) | 20.5(17, 28) | 23(20, 33) | 0.133 |
| RV pressure after repair (mmHg) | 40(35, 51) | 40(34, 50) | 40(35, 55) | 0.713 |
| Systemic pressure after repair (mmHg) | 76(69, 86) | 78(70, 88) | 76(68, 85) | 0.353 |
| RVSP/LVSP | 0.52(0.44, 0.67) | 0.50(0.43, 0.62) | 0.54(0.44, 0.71) | 0.271 |
| CVP (mmHg) | 8(7, 10) | 8(7, 10) | 8 (7, 10) | 0.791 |
| TAP (n) | 59(44.4%) | 23(37.1%) | 36(50.7%) | 0.117 |

PDA: patent ductus arteriosus; LVEDVI: left ventricular end-diastolic volume index; RVEDVI: right ventricular end-diastolic volume index;; LVSP: left ventricle systolic pressure; RVSP: right ventricle systolic pressure; CVP: central venous pressure; TAP: transannular patch; APC: Aortopulmonary collaterals; CSA: cross-sectional area; PA: pulmonary artery; RV: right ventricle;; Hgb: hemoglobin;

**~~Supplemental~~** **~~Table 5: Comparison of in-hospital outcome between low and high aortopulmonary collaterals density~~**

| **~~Variable~~** | **~~Sum (n=133)~~** | **~~Low APC density(APC-CSA <3.0 mm~~^~~2~~^~~/m~~^~~2~~^~~, n=62)~~** | **~~High APC density (APC-CSA ≥3.0mm~~^~~2~~^~~/m~~^~~2~~^~~, n=71)~~** | ***~~P~~*~~- value~~** |
| --- | --- | --- | --- | --- |
| **~~Composite outcome (n)~~** | ~~41(30.8%)~~ | ~~12 (19.4%)~~ | ~~29(40.8%)~~ | ~~0.007~~ |
| ~~Death (n)~~ | ~~3(2.3%)~~ | ~~1(1.6%)~~ | ~~2(2.8%)~~ | ~~0.642~~ |
| ~~Circulatory support (n)~~ | ~~1(0.7%)~~ | ~~1(1.6%)~~ | ~~0（0%）~~ | ~~0.225~~ |
| ~~Cardiac arrest (n)~~ | ~~5(3.8%)~~ | ~~1(1.6%)~~ | ~~4(5.6%)~~ | ~~0.284~~ |
| ~~Renal insufficiency (n)~~ | ~~5(3.8%)~~ | ~~2(3.2%)~~ | ~~3(4.2%)~~ | ~~0.741~~ |
| ~~Hepatic insufficiency (n)~~ | ~~36(27.1%)~~ | ~~11(17.7%)~~ | ~~25(35.2%)~~ | ~~0.024~~ |
| ~~Lactic acidosis (n)~~ | ~~27(20.3%)~~ | ~~12 (19.4%)~~ | ~~15(16.9%)~~ | ~~0.080~~ |
| **~~Delayed chest closure (n)~~** | ~~6(4.5%)~~ | ~~1(1.6%)~~ | ~~5(7.0%)~~ | ~~0.121~~ |
| **~~Maximal VIS in the first 24 hours after ICU admission~~** | ~~9(5,20)~~ | ~~7(3, 18)~~ | ~~10(6, 20)~~ | ~~0.008~~ |
| **~~Duration of vasoactive support (hr)~~** | ~~77(40,142)~~ | ~~48(23, 139)~~ | ~~96(61, 161.5)~~ | ~~0.010~~ |
| **~~Ventilation time (hr)~~** | ~~20(5,91)~~ | ~~11(4, 67)~~ | ~~30(6, 119)~~ | ~~0.042~~ |
| **~~ICU duration (day)~~** | ~~5(3,8)~~ | ~~4(2,7)~~ | ~~6(3,9)~~ | ~~0.014~~ |
| **~~Hospital stay (day)~~** | ~~9(7,13)~~ | ~~8(7, 12)~~ | ~~10(8, 15)~~ | ~~0.027~~ |
| **~~Prolonged chest insertion (n)~~** | ~~35(26.3%)~~ | ~~10(16.1%)~~ | ~~25(35.2%)~~ | ~~0.013~~ |
| **~~Postoperative catheterization (n)~~** | ~~7(6.0%)~~ | ~~2(3.2%)~~ | ~~6(8.5%)~~ | ~~0.208~~ |

~~APCs: aortopulmonary collaterals; CSA: Cross-sectional Area; VIS: vasoactive-inotropic score~~

**Supplemental Table ~~6~~5: Cause and outcome of patients underwent postoperative catheterization**

| **Patient Number** | **Age** | **Weight**  **(kg)** | **Indexed APC-CSA(mm^2^/m^2^)** | **Nakata index(mm^2^/m^2^)** | **LVEDVI (ml/m^2^)** | **Cause of Catheterization** | **LAP (mmHg)** | **Procedure** | **Outcome** |
| --- | --- | --- | --- | --- | --- | --- | --- | --- | --- |
| 89 | 35.9 years | 34 | 3.21 | 188.8 | 23.7 | Prolonged ventilation | 23 | Coiled four enlarged APCs | Alive |
| 22 | 19.7 months | 8 | 10.1 | 94.1 | 46.3 | Prolonged ventilation, bloody airway secretion, and bilateral chest effusion | 30 | Coiled six enlarged APCs | Alive |
| 151 | 6.4 months | 7.5 | 2.12 | 79.7 | 35.1 | Failed to come off bypass due to high RV pressure, patient was on ECMO | Not measured | Bilateral PA stenting | Alive |
| 18 | 12.3 months | 8.5 | 1.91 | 98.9 | 43.9 | Low cardiac output, respiratory failure | 14 | Coiled two enlarged APCs | Alive |
| 43 | 6.7 months | 7.2 | 4.15 | 93.9 | 48.6 | Failure of extubation, right chest effusion | 21 | Coiled three enlarged APCs | Alive |
| 137 | 44.3 months | 10.5 | 3.46 | 168.4 | 41.2 | Low cardiac output | 20 | Coiled three enlarged APCs | Alive |
| 145 | 8.8 months | 8.5 | 3.84 | 99.9 | 45.0 | Failure of extubation, right chest effusion | 27 | Coiled three enlarged APCs | Alive |
| 148 | 22.7 months | 9 | 3.42 | 249.7 | 42.2 | Reintubation | 25 | Coiled three enlarged APCs | Alive |

APCs: aortopulmonary collaterals; CSA: Cross-sectional Area; LVEDVI: Left ventricle end-diastolic volume index; LAP: left atrium pressure

**SUPPLEMENTAL FIGURE LEGENDS**

**Supplemental Figure 1: Representative images of aortopulmonary collaterals in Tetralogy of Fallot with pulmonary stenosis**

The site of the aortopulmonary collaterals(APCs) ostium(star) were coded as orthotopic when the arteries were originating from the descending aorta between the levels of the T5 and T6 vertebrae**(A)** or ectopic when identified at a level of the descending aorta other than the expected origin (ie, outside levels T5-T6), such as the level of the aortic arch**(B)** or from any aortic branch vessels(**C);** The origin of each APC was reported as intercostal trunk, common trunk, or directly from the aorta**(D,** black arrow**)**. A common trunk (black arrow) was defined as arising of the right and left APCs(black triangle) as a common trunk from the descending aorta**(E)**; Arising of right or left APCs(black triangle) and an intercostal artery(white arrow) from the descending aorta as a common trunk was defined as an intercostal trunk **(F**, black arrow).

**Supplemental Figure 2:** **The correlation between indexed proximal and distal aortopulmonary collaterals cross-sectional area**

Indexed distal aortopulmonary collaterals cross-sectional area (APC-CSA) was significantly correalted with indexed proximal APC-CSA (r=0.47, p=0.00)

**Supplemental Figure 3: The cutoff point of the indexed total distal aortopulmonary collaterals cross-sectional area for the occurrence of composite outcome**

The area under the ROC curve for predicting the occurrence of in-hospital composite outcome was 0.77(95% confidence interval: 0.68-0.86)(p=0.000). The cutoff point(arrow) of the indexed total distal aortopulmonary collaterals cross-sectional area was ≥ 3.0mm^2^/m^2^ with a sensitivity of 73%, specificity of 71%.
